# Application of the dynamic needle tip positioning method for ultrasound-guided arterial catheterization in elderly patients: A randomized controlled trial

**DOI:** 10.1371/journal.pone.0273563

**Published:** 2022-08-26

**Authors:** Jae-Geum Shim, Eun A. Cho, Tae-Ryun Gahng, Jiyeon Park, Eun Kyung Lee, Eun Jung Oh, Jin Hee Ahn

**Affiliations:** 1 Department of Anesthesiology and Pain Medicine, College of Medicine, Graduate School, Kyung Hee University, Seoul, Korea; 2 Department of Anesthesiology and Pain Medicine, Kangbuk Samsung Medical Center, Sungkyunkwan University School of Medicine, Seoul, Republic of Korea; 3 Department of Anesthesiology and Pain Medicine, Samsung Medical Center, Sungkyunkwan University School of Medicine, Seoul, Republic of Korea; Universita degli Studi Magna Graecia di Catanzaro, ITALY

## Abstract

**Background:**

Arterial cannulation in elderly patients is difficult because of age-related morphological changes. Applying dynamic needle tip positioning (DNTP) that guides the catheter to position inside the vessel sufficiently may aid in successful cannulation.

**Methods:**

This prospective study enrolled patients aged over 70 years, who were scheduled for elective surgery under general anaesthesia with arterial cannulation. The patients were randomly assigned to the DNTP (group D, n = 76) or the conventional short-axis view(group C, n = 75) group. The arterial depth, diameter, and arterial conditions(calcification, segmental stenosis, and tortuosity) were evaluated using ultrasound, before puncture. We recorded the first attempt success, cannulation time, the number of attempts, and cannulation-related complications.

**Results:**

A total of 151 patients were enrolled in this study. The first attempt success rate in group D was significantly higher than that in group C (89% versus 72%; *P = 0*.*0168*). The median cannulation time per last attempt in group D versus group C was 25 versus 30 sec(*P = 0*.*0001*), and the overall cannulation time was 25 versus 35 sec(*P = 0*.*0001*), respectively. Arterial cannulation per last attempt and overall cannulation time were shorter in group D. The number of attempts was higher in group C (*P = 0*.*0038*). The occurrence rate of hematoma was significantly lower in group D (16% versus 47%, relative risk = 3.0, *P = 0*.*0001*).

**Conclusions:**

The DNTP method may improve the first attempt success rate of arterial cannulation and reduce complications in elderly patients over 70 years of age.

## Introduction

Arterial catheterization is performed to monitor blood pressure continuously and conduct frequent blood laboratory tests during operation period [1]. Different sites can be used for arterial catheterization, among which, the radial artery is the most commonly selected site, since it is located on the surface and has a low complication rate [2].

Recently, the placement of a catheter into the radial artery by palpation of the arterial pulse is changing to an ultrasound-guided method, in which the positioning of the catheter and artery is visually confirmed by ultrasound [3–5]. In particular, the dynamic needle tip positioning (DNTP) method, which repeatedly confirms the position of the tip of Angiocatheter needle in the centre of the intra-arterial lumen, increases the success rate of arterial catheterization and decreases the incidence of catheterization-related complications [6–9]. In previous studies, the first attempt success rate of the DNTP method was about 85–94%, whereas the conventional ultrasound short axis view method showed various success rates of 50%-80% [8, 10–12]. As age increases, it gets difficult to predict the running of blood vessels due to vascular changes, such as atherosclerotic and tortuous changes [13–15]. In patients with vascular morphologic changes, using the DNTP method to guide the catheter to be sufficiently positioned inside the blood vessel may be helpful for safe and successful cannulation.

Therefore, we conducted a randomized controlled trial to evaluate the efficacy of the ultrasound guided DNTP method in elderly patients over 70 years of age. We hypothesised that the DNTP technique could increase the first attempt success rate and decrease catheterization-related complications compared to the conventional short-axis view method. The primary outcome of this study was the success rate of arterial cannulation in the first attempt. The secondary outcomes were cannulation time, the number of insertion attempts, and catheterization-related complications.

## Material and methods

This prospective study was performed at Kangbuk Samsung Medical Centre, Seoul, Korea. This study was approved by the Kangbuk Samsung medical centre Institutional Review Board (IRB No. 2019-04-020) and written informed consent was contained from all subjects participating in the trial. The trial was registered prior enrolment at the Clinical Trials of Korea (KCT 0004232, Principle investigator Jin Hee Ahn, Date of registration Aug 21/2019). Patients aged > 70 years with ASA physical status II-IV, scheduled for elective surgery, between October 2019 and June 2020, under general anesthesia with arterial cannulation were enrolled. The exclusion criteria were patients with an abnormal Allen test, malformation of the forearm arteries on ultrasound, skin erosions, haematomas, unstable vital signs, or emergency surgery.

### Randomization and blinding

All patients were randomly assigned to one of two groups. In Group C, catheterization was performed in a short-axis view using the conventional method, and in group D, catheterization was performed using the DNTP method. The statistical team generated a randomized list through web program(http://www.randomizer.org). A list of assigned groups was placed in an opaque envelope and delivered to the investigator immediately prior to arterial catheterization. Two experienced anesthesiologists (JHA and JS) performed this study, alternating arterial catheterization and pre and post evaluation for radial artery. Prior to the start of this study, the two investigators performed arterial condition evaluation more than 100 times together, and practiced the DNTP method and the conventional method more than 200 times. One investigator was arterial catheterization performer, catheterization was performed according to the assigned group, and immediately before the procedure, she (or he) entered the operating room and was blinded to the results of the evaluation of the artery condition. The other investigator performed an assessment of the radial artery condition before catheterization and complications after catheterization using ultrasonography (US), at the time of catheterization, the investigator left the operating room and was blinded to which group the patient was. The results of both investigators were recorded and managed by authors who did not participate in this study, and were collected and analyzed only after all studies were completed.

### Anesthesia protocol

The patients did not receive any premedication. Routine monitoring, such as electrocardiography, pulse oximetry, non-invasive blood pressure, and BIS (bispectral index), was performed on the patients upon arrival to the operating room. For anesthesia induction, intravenous anesthetics were administered, such as propofol (1.5 mg/kg), rocuronium (0.6 mg/kg), and remifentanil (1 mcg/kg). Endotracheal intubation was performed after muscle relaxation was confirmed with a twice-train-of-four (TOF) count of zero. After confirmation of successful tracheal intubation, anesthesia was maintained with sevoflurane (1.5%–2%) and remifentanil (0.05–0.2 μg/kg/min), and radial artery cannulation was performed.

### Evaluation for radial artery condition

The patient’s arm was slightly abducted, and the wrist joint was positioned in an extended manner by fixing a wrist pad (CAS, SHmedical Co. Ltd., Korea) under the wrist. Before arterial catheterization, the evaluation investigator (EAC) evaluated the initial radial artery condition using an ultrasound machine (GE Healthcare, VenuGo^TM^, Milwaukee, WI, USA) with an L25x/13-6 MHz transducer (linear type).

A point, 1 cm away from the wrist crease, was marked with a surgical pen as the needle insertion site (P0, skin puncture site). Mark P1 (radial artery puncture site) at a point 0.5 cm proximal from P0 and mark P2 at a point 2 cm proximal to P1 were also marked. The depth and diameter of the radial artery at each location (P1 and P2) were measured using an ultrasound calliper. In the short-axis view, the depth was measured from the skin to the radial artery anterior wall. The diameter was measured as the distance from the radial intra-arterial anterior wall to the posterior wall.

Evaluation of radial artery conditions consisted of calcification, segmental stenosis, and tortuosity. The calcification score was 1, when calcification was observed in the artery while tracing the ultrasound probe between P1 and P2. Segmental stenosis was evaluated by tracing between P1 and P2 in a short-axis view, and a score was assigned according to the degree of stenosis compared to the diameter of P1. If the degree of stenosis demonstrated a decrease in diameter between P1 and P2 of less than 10%, 0 points were given;1 point was given for a decrease in diameter of 10%-49%; 2 points were given if the decrease in diameter were 50% or more. Tortuosity was assessed by placing the ultrasound probe parallel to the vessel between points P1 and P2. In the ultrasound long axis view, if the vessel is straight, score 0, if the tortuous vessel is observed up-down or left- right, 1 point, and if the tortuous vessel is observed up-down and left-right, 2 points are given. The total score is distributed on a scale from 0 to 5 according to degree of radial artery morphologic change.

### Placement of arterial catheterization

Arterial cannulation was performed by ultrasound-guided radial artery puncture using ultrasound with a linear probe and a 20-gauge catheter (Angiocatheter, Sewoon Medical Ltd, Korea). The ultrasound probe, in a sterile cover, was positioned on the skin surface, and the radial artery was visualised in the short-axis view. The probe position was adjusted so that the cross-section of the radial artery was centered on the screen.

#### Conventional ultrasound group (Group C)

**Fig 1:**
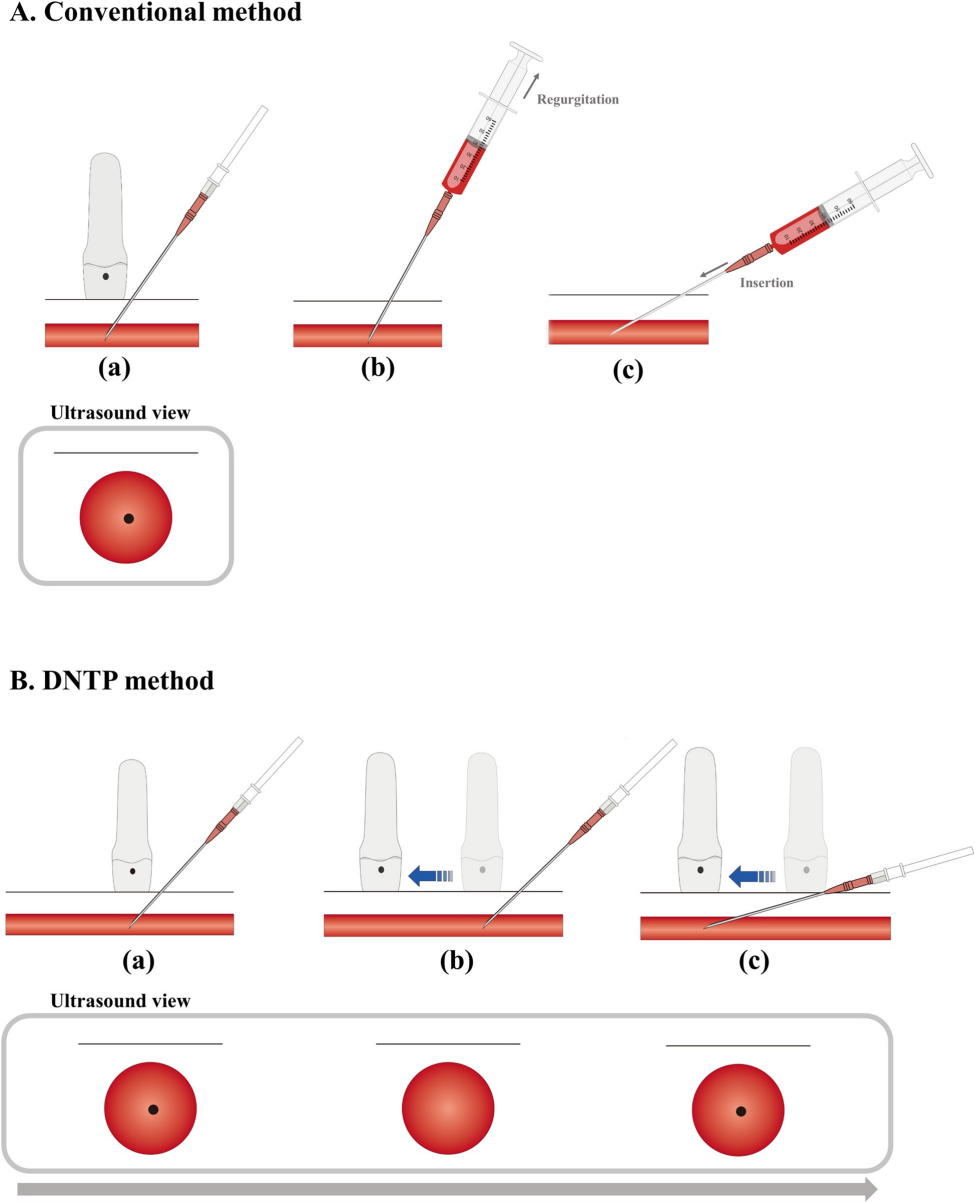
Illustrations for two ultrasound-guided radial artery cannulation methods. **A. Conventional method** (a) Confirmation of needle tip visualization in the centre of intra-arterial lumen on the ultrasound screen. After confirming that blood has been aspirated into the catheter hub, the needle is advanced slightly whilst reducing the puncture angle, and an external catheter was inserted into the artery. Then, after the inner guide needle is removed, the (b)and (c) process are performed sequentially if the blood return is not observed. (b) The outer catheter was withdrawn by applying negative pressure until the blood aspiration is observed again, (c)If blood aspiration continued, an outer- catheter is inserted into the artery. **B. DNTP (dynamic needle tip positioning) method** (a) The skin and radial artery are punctured, and the tip of the needle is seen in the centre of the intra-arterial lumen. (b) The probe is moved forward slightly until the needle tip disappears in the intra-arterial lumen. (c) The needle tip is slightly advanced tip of the needle. Repeat steps (a)-(c) 2 or more times to ensure that the entire catheter of the outer cannula is advanced into the artery.

The needle was inserted into the skin 5 mm from the center of the probe at an angle of approximately 30° to 45°. Without moving the ultrasound probe, Angiocatheter was advanced under ultrasound guidance until it was confirmed that the tip of the needle placed the center of the artery (Fig 1A(a)). After confirmation, blood was aspirated into the catheter hub, the needle was advanced slightly whilst reducing the puncture angle, and an external catheter was inserted into the artery. However, after the inner guide needle was removed, the authors considered a posterior wall puncture of the artery if the blood return was not observed. The outer catheter was withdrawn by applying negative pressure to the syringe until blood aspiration was observed again. If blood aspiration continued, an outer catheter was inserted into the artery (Fig 1A(b) and 1A(c)). If blood aspiration to the syringe was not confirmed, even after the catheter was carefully withdrawn, it was considered a failure. A reattempt was performed in the same artery using the same method.

#### Dynamic needle tip positioning group (Group D)

The DNTP method is shown in Fig 1B. The needle was inserted into the skin 5 mm from the center of the probe at an angle of approximately 30° to 45°. The needle tip was visualized as a small bright dot in the center of the artery cross-section, on the ultrasound screen (Fig 1B(a)). The ultrasound probe was slightly moved in the proximal direction until the needle tip disappeared (Fig 1B(b)). The Angiocatheter was advanced until it appeared again in the center of the radial artery (Fig 1B(c)). This process was repeated two or more times. After confirming that both the needle and catheter were placed at least 1 cm inside the arterial lumen [8], the outer catheter was placed inside the radial artery, and the guided needle was removed.

## Outcome measurements

The time to cannulation per last attempt was defined as the time from the start of ultrasound scanning on the last attempt (final success attempt) to the appearance of an arterial waveform on the monitor. The time to overall cannulation was defined as the time from the start of ultrasound scanning during the first attempt to the appearance of an arterial waveform on the monitor. If cannulation was not successful with the assigned method after 10 min, it was defined as cannulation failure. The study was stopped, and the operators were free to use any cannulation method.

The collected data included the first-attempt success of radial arterial line catheterization, number of cannulation attempts, time to cannulation per last attempt, and overall cannulation time. Haemodynamic variables included heart rate, systolic blood pressure, and diastolic pressure before skin puncture. Patient characteristics were obtained from the electronic medical records.

Arterial cannulation-related complications were defined as follows: haematoma was defined as a newly localised echogenic fluid collection along or adjacent to the arterial wall and thrombosis was defined as a newly developed blood clot in the radial artery [16]. Thrombosis was confirmed when a clot in the radial artery was detected by ultrasound [16]. Vasospasms were considered to have occurred if the radial artery lumen narrowed by more than 50% in diameter as demonstrated by ultrasound [6]. These outcomes were assessed and recorded immediately after cannulation by those who did not participate in the study.

### Statistical analysis

A pilot study was conducted with 51 patients to calculate the sample size. The first attempt success rate was 92.3%(24/26) in group D and 72%(18/25) in group C. Based on this, sample size calculation was performed bilaterally using a Fisher’s exact test (α err = 0.1, Power (1-β err) = 0.9). The sample size was 69 in each group, and a total of 152 patients were planned, considering a dropout rate of 10%. Data are presented as number (percentage, %) or mean±standard deviation (SD) or as median (interquartile range, IQR), as appropriate. Relative risks and 95% confidence intervals (CIs) were quoted when necessary. Relative risk and 95% CIs excluding 1 were considered statistically significant. Differences and 95% CIs excluding 0 were considered statistically significant. Continuous variables were compared using the t-test or Wilcoxon signed-rank test, and the Shapiro–Wilk test was used to test for normality. As appropriate, categorical variables were analysed using Pearson’s chi-square test or Fisher’s exact test. The overall cannulation time was compared using the Kaplan–Meier cumulative incidence plot with log-rank tests. All statistical analyses were performed using MedCalc® Statistical Software version 20.014 (MedCalc Software Ltd, Ostend, Belgium; https://www.medcalc.org; 2021). Statistical significance was set at *p* <0.05.

## Results

**Fig 2:**
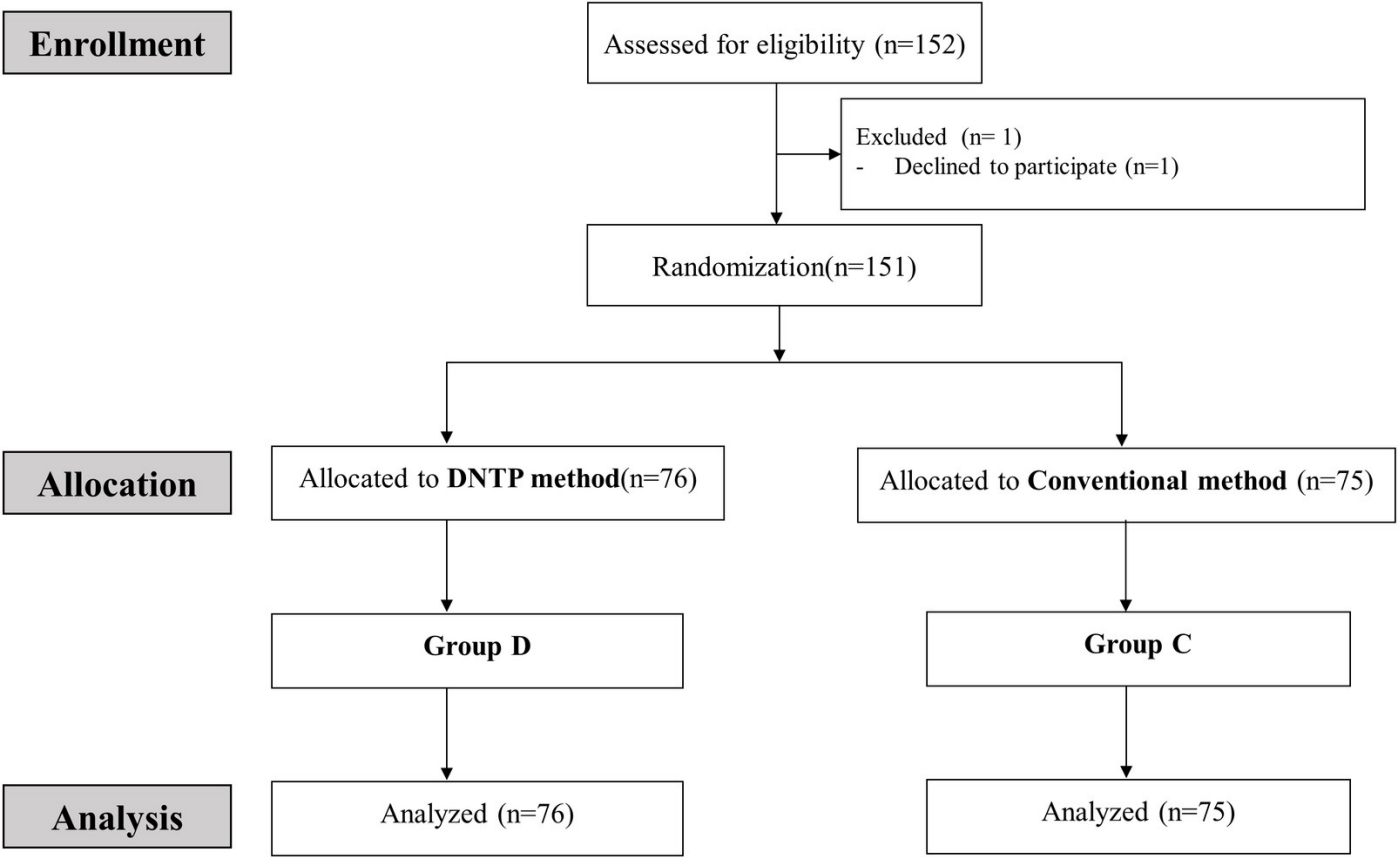
CONSORT flow diagram.

A total of 152 patients were assessed for eligibility (Fig 2). Of these, 1 patient was excluded because they declined to participate (n = 1). Therefore, 151 patients were enrolled and assigned to one of two groups: Group D (n = 76) and Group C (n = 75). No patients dropped out after allocation of the study. Baseline demographic characteristics did not differ significantly between the two groups (Table 1). The characteristics and conditions of the radial artery are presented in Table 1. Depth from the skin to the artery, inner diameter of the artery, and radial artery condition score were not significantly different between groups D and C.

**Table 1:** Patient characteristics.

	Group D (n = 76)	Group C (n = 75)
Sex, n (Female / Male)	28 / 48	38 / 37
Age, year	76 ± 5	75 ± 5
Weight, kg	61 ± 9	61 ± 10
Height, cm	158 ± 9	158 ± 9
BMI		
ASA PS (II / III / Ⅳ)	39 / 36 / 1	29 / 45 / 1
Underlying disease		
DM	38	30
HTN	22	22
Renal disease	4	5
CVA	7	11
Cardiac disease	16	17
Anticoagulant therapy		
(aspirin / clopidogrel / warfarin / etc.)	14 / 5 / 0 / 0	16 / 5 / 1 / 2
Radial artery puncture History	29 (38)	20 (27)
Heart rate (per min)	77 ± 15	77 ± 15
Blood pressure (D / S, mmHg)	77 ± 13/144 ± 21	76 ± 15 / 141± 23
**Evaluation for radial artery**		
Depth from skin to artery, mm		
Distal (P1)	2.4 ± 0.8	2.5 ± 1.0
Proximal (P2)	2.6 ± 1.2	2.8 ± 1.2
Inner diameter of artery, mm		
Distal (P1)	2.5 ± 0.5	2.6 ± 0.5
Proximal (P2)	2.5 ± 0.6	2.5 ± 0.5
**Radial artery condition score**		
Calcification (0 / 1)	39 / 37	37 / 38
Stenosis (0 / 1 / 2)	39 / 23 / 14	40 / 22 / 13
Tortuosity (0 / 1 / 2)	21 / 20 / 35	22 / 18 / 35
Total score	2 [1,4]	2 [1,4]

Data are presented as the median[IQR], mean ± SD, or number.

Abbreviation: ASA PS, American Society of Anaesthesiologists Physical Status; DM, diabetes mellitus; BMI, body mass index; HTN, Hypertension; CVA, cerebrovascular accident

### Primary outcome

**Fig 3:**
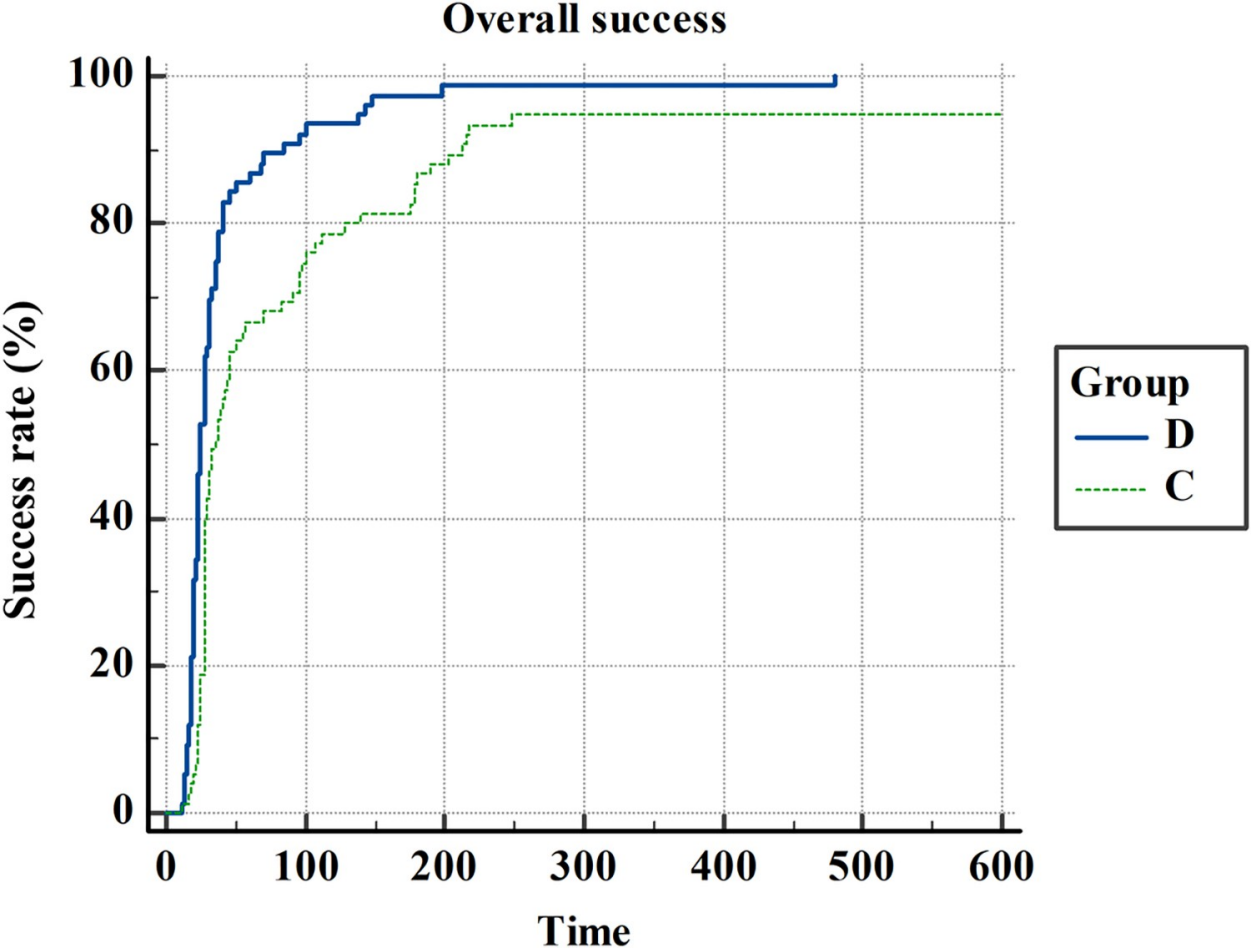
Kaplan–Meier curves for the overall catheterization success time. **D:** Group D, dynamic needle tip positioning method. **C**: Group C, conventional short-axis method. Time(x-axis) is defined as the time from the start of ultrasound scanning during the first attempt to the appearance of an arterial waveform on the monitor. If cannulation was not successful with the assigned method after 10 min, it was defined as cannulation failure.

The results of the study are shown in Table 2. The first attempt success rate in group D was significantly higher than group C [89% versus 72%; the proportional difference (95% CI), 17.0 (3.0 to 31.2), *P = 0*.*0168*]. The number of overall successes within 10 min in Group D and Group C was 76 (100%) and 71 (95%), respectively, and there was no significantly different between two groups. [the proportional difference (95% CI), 5.3 (-0.5 to 13.2), *P = 0*.*0584*]. Kaplan–Meier curves for the overall cannulation success time are shown in Fig 3. There are significant differences between two groups(Log-lank: *P<0*.*0001*).

**Table 2:** Study results.

	Group D (n = 76)	Group C (n = 75)	P	Proportion differences or Median difference (95%CI)
First attempt success, n (%)	68 (89)	54(72)	0.0168 ^a^	17.0 (3.0 to 31.2)^a^
Overall success within 10 min, n (%)	76 (100)	71(95)	0.0584 ^b^	5.3 (-0.5 to 13.2) ^b^
Cannulation time per last attempt, sec	25 [20, 35]	30[27, 47]	0.0001 ^c^	7.0 (4.0 to 11.0)^c^
Overall cannulation time, sec	25 [20, 37]	32[27, 94]	0.0001 ^c^	9.0 (5.0 to 14.0)^c^
Number of attempts, n (%)			0.0038 ^d^	-
1	68(89)	54(72)	-	-
2	6(8)	9(12)	-	-
3	1(1)	9(12)	-	-
≥4	1(1)	3(4)	-	-

Data are presented as number (percentage,%) and median[Inter Quartile Range]

^a^*P* value and proportional differences (95% CI) are calculated from the χ2 test.

^*b*^*P* value and proportional differences (95% CI) are calculated from the Fisher exact test.

^*c*^*P* values and median differences (95% CI) were calculated using the Mann–Whitney U test.

^*d*^*P* value is calculated from the χ2 test for trend.

### Secondary outcome

The median [interquartile range] cannulation time per last attempt in group D vs group C was 25 [20,35] versus 30 [27, 47] sec (*P = 0*.*0001*), and the overall cannulation time was 25 [20, 37] versus 35 [27, 100] sec (*P = 0*.*0001*), respectively. Arterial cannulation per last attempt and overall cannulation time were shorter in group D (*P = 0*.*0001*, respectively). The observed interquartile range was wider for group C. The number of attempts tended to be higher in group C (*P = 0*.*0038*).

Arterial cannulation-related complications are presented in Table 3. Haematoma occurred in 16% of group D and 47% of group C [relative risk (95% CI), 3.0 (107–5.2), P = 0.0001]. Thrombosis and vasospasm had higher incidence rate in group C, but the difference was not statistically significant. No severe complications related to radial artery cannulation, such as pseudoaneurysm formation or radial artery occlusion, occurred in any patient until discharge.

**Table 3:** Cannulation related complications.

Complications, n(%)	Group D (n = 76)	Group C (n = 75)	P	*Relative risk (95% CI)
Hematoma	12 (16)	35 (47)	0.0001^e^	3.0 (1.7–5.2)
Thrombosis	1 (1)	5 (7)	0.1163^f^	2.1 (0.6–42.3)
Vasospasm	2 (3)	7 (9)	0.0830 ^f^	3.5 (0.8–16.5)

Data are presented as number (percentage,%)

^e^*P-*value and relative risk (95%CI) are calculated from the χ2 test.

^f^*P* value and relative risk (95%CI) is calculated from Fisher exact test

*Relative risk is calculated in group C compared to group D.

## Discussion

The current study demonstrates that the ‘DNTP’ method improved the first attempt success rate of radial arterial cannulation in elderly patients over 70 years of age, reduces the overall cannulation time, and reduces the incidence of cannulation-related complications. To the best of our knowledge, this is the first study to investigate the usefulness of the DNTP method in elderly patients.

Successful cannulation of an artery requires completing two separate steps: puncturing the arterial wall and subsequent catheter advancement. The DNTP method has the advantage of checking the anterial arterial wall puncture in the short-axis view and visually confirming the catheter advancement whilst confirming that the needle tip is positioned at the centre of the artery cross-section [17–19]. In patients who underwent bilateral radial artery catheterization, the first attempt success rates of the DNTP and traditional palpation techniques were 95% and 57.5%, respectively [5]. In neonates, the first attempt success rate of arterial catheterization in the DNTP was higher than in the palpation methods (40% vs 10%) [16]. In addition, comparing the ultrasound-guided long-axis view (in-plane) method with DNTP method, the first attempt success rate was 94% versus 68%, indicating that the DNTP method was superior [12]. In a study comparing conventional ultrasound-guided short-axis views (out-of-plane) with DNTP method, in children under 3 years of age at risk of difficult radial artery cannulation (depth of radial artery >4 mm), the first attempt success rate(85% versus 50%) and the overall success rate(95% versus 60%) were significantly higher in DNTP method group [11]. Therefore, the DNTP method is more efficient in clinical practice than traditional palpation and conventional ultrasound-guided methods (short-and long-axis views).

The strength of our study is that we included elderly patients over 70 years of age as the study population. Arterial cannulation in elderly patients is challenging due to age-related changes, such as segmental stenosis, atherosclerotic changes, and tortuosity of blood vessels. Vascular changes such as tortuosity increased linearly with age, and these morphological and anatomical changes affected the radial artery catheterization difficulty (odds ratio = 17.8) [13, 20, 21]. In our ultrasound radial artery evaluation, one or more morphologic changes of the radial artery such as calcification, segmental stenosis, and tortuosity were observed in 70% of patients. Thus, the DNTP method is more effective than the conventional method for radial artery cannulation in elderly patients.

The inner guide needle is removed immediately after the anterior wall puncture in the conventional method. Only the soft outer catheter slides into the inside of the blood vessel, which has the advantage of less damage to the inner wall of the blood vessel. Compared to the inner-guide needle, the outer catheter has a larger diameter and soft material. It is difficult for the catheter to advance into the blood vessel with severe calcification of the vessel wall, segmental stenosis, or acute angle tortuosity. In contrast, the DNTP method can damage the inner arterial wall whilst the inner-guide needle moves inside the blood vessel. However, even if there is tortuosity or segmental stenosis, it is possible to visually confirm that the catheter is located at the centre of the blood vessel. The cannulation success rate is increased because the outer catheter can be mounted with a sufficient length inside the vessel. In addition, in our results, the incidence of complications related to arterial injury, such as haematoma, thrombosis, and vasospasm, were lower in the group D. Therefore, the DNTP method is relatively safe and useful for elderly patients.

Our study has several limitations. First, the DNTP technique depends on the skill of the operator. In our study, two experienced operators (>200) performed the procedure to reduce the error range of the results. However, in the previous DNTP method study, each group’s first attempt success rate was more than 80%, even in operators with different experience values consisting of residents, fellows, and faculty members [8]. Second, two investigators (JHA and JS) alternately performed different roles blindly, preventing them from knowing each other’s examination or catheterization results. But, since the principal investigator (JHA) who planned this study is one of the investigators who conducted catheterization, the possibility that it may affect the study results cannot be excluded. Third, we did not include the correlation between the radial artery condition score and radial artery cannulation difficulty in main results because there was a concern that the focus of our study results may be confused. It was used only as a tool to confirm that there was no difference in the evaluation of the radial artery condition between the two groups. Further studies are needed to confirm the degree of cannulation difficulty according to the radial artery conditions.

## Conclusions

The DNTP method tended to show a higher first-attempt success rate, a reduced number of attempts, and a faster time to catheterization for elderly patients. Ultrasound-guided dynamic visualization of the needle tip is helpful when applied during arterial cannulation in patients with age-related arterial changes.

## Supporting information

S1 Checklist (DOC)

S1 Data (XLSX)

S1 File (DOCX)
