# Supplementary material for: Application of the dynamic needle tip positioning method for ultrasound-guided arterial catheterization in elderly patients: A randomized controlled trial
Source: PLoS One. 2022 Aug 26;17(8):e0273563. doi: 10.1371/journal.pone.0273563 (PMC9417032; doi:10.1371/journal.pone.0273563)
Supplement: S1 File — (DOCX) [file pone.0273563.s003.docx]

# Research Protocol

#### Project summary

Recently, the placement of a catheter into the radial artery by palpation of the arterial pulse is changing to an ultrasound-guided method that visually confirms the placement of the catheter in the artery using ultrasound. In particular, the dynamic needle tip positioning (DNTP) method, which repeatedly confirms the position of the tip of Angiocatheter needle in the centre of the intra-arterial lumen, increases the success rate of arterial catheterization and decreases the incidence of catheterization-related complications. As age increases, it gets difficult to predict the running of blood vessels due to vascular changes, such as atherosclerotic and tortuous changes. In patients with vascular morphologic changes, using the DNTP method to guide the catheter to be sufficiently positioned inside the blood vessel may be helpful for safe and successful cannulation.

Therefore, we conducted a randomised controlled trial to evaluate the efficacy of the ultrasound guided DNTP method compared to conventional short-axis view method in elderly patients over 70 years of age.

#### General information

**Application of the dynamic needle tip positioning method for arterial catheterisation in elderly patients over 70 years of age: a randomised controlled trial**

- **Investigator**
- Jin Hee Ahn
- Jae-Geum Shim
- **Principal Investigator**

**Jin Hee Ahn,** M.D., Ph.D.

Tel.: +82-2-2001-2321; Fax: +82-2-2001-2461; E-mail: [blatt.ahn@samsung.com](mailto:blatt.ahn@samsung.com)

- **Institution**

Department of Anesthesiology and Pain Medicine, Kangbuk Samsung Medical Center, Sungkyunkwan University School of Medicine, Seoul, Korea

#### Rationale & background information

#### Arterial catheterisation is performed to monitor blood pressure continuously and conduct frequent blood laboratory tests during operation period. [1] Different sites can be used for arterial catheterization, among which, the radial artery is the most commonly selected site, since it is located on the surface and has a low complication rate. [2]

Recently, the placement of a catheter into the radial artery by palpation of the arterial pulse is changing to an ultrasound-guided method that visually confirms the placement of the catheter in the artery using ultrasound.[3] In particular, the dynamic needle tip positioning (DNTP) method, which repeatedly confirms the position of the tip of angiocatheter needle in the centre of the intra-arterial lumen, increases the success rate of arterial catheterisation and decreases the incidence of catheterisation-related complications.[4-6] As age increases, it gets difficult to predict the running of blood vessels due to vascular changes, such as atherosclerotic and tortuous changes.[7-9] In patients with vascular morphologic changes, using the DNTP method to guide the catheter to be sufficiently positioned inside the blood vessel may be helpful for safe and successful cannulation.

#### References

1. Shiloh, A.L., et al., *Ultrasound-guided catheterization of the radial artery: a systematic review and meta-analysis of randomized controlled trials.* Chest, 2011. **139**(3): p. 524-529.

2. Sandhu, N.S. and B. Patel, *Use of ultrasonography as a rescue technique for failed radial artery cannulation.* J Clin Anesth, 2006. **18**(2): p. 138-41.

3. Tang, L., et al., *Ultrasound guidance for radial artery catheterization: an updated meta-analysis of randomized controlled trials.* PLoS One, 2014. **9**(11): p. e111527.

4. Seto, A.H., et al., *Real-time ultrasound guidance facilitates transradial access: RAUST (Radial Artery access with Ultrasound Trial).* JACC Cardiovasc Interv, 2015. **8**(2): p. 283-291.

5. Peters, C., et al., *Ultrasound guidance versus direct palpation for radial artery catheterization by expert operators: a randomized trial among Canadian cardiac anesthesiologists.* Can J Anaesth, 2015. **62**(11): p. 1161-8.

6. Kiberenge, R.K., K. Ueda, and B. Rosauer, *Ultrasound-Guided Dynamic Needle Tip Positioning Technique Versus Palpation Technique for Radial Arterial Cannulation in Adult Surgical Patients: A Randomized Controlled Trial.* Anesth Analg, 2018. **126**(1): p. 120-126.

7. Ostojić, Z., et al., *Frequency of radial artery anatomic variations in patients undergoing transradial heart catheterization.* Acta Clin Croat, 2015. **54**(1): p. 65-72.

8. Barbeau, G.R., *Radial loop and extreme vessel tortuosity in the transradial approach: Advantage of hydrophilic-coated guidewires and catheters.* Catheterization and Cardiovascular Interventions, 2003. **59**(4): p. 442-450.

9. Wessel, E., et al., *Quantification of the distal radial artery for improved vascular access.* Folia Morphol (Warsz), 2015. **74**(1): p. 100-5.

#### Study goals and objectives

#### To evaluate the efficacy of the ultrasound guided DNTP method in elderly patients over 70 years of age.

#### Study Design

- **Design** : Prospective randomized conrtrolled trial
- **Research population** : elderly patients over 70 years of age
- **Inclusion criteria**
- Patients aged > 70 years with ASA physical status II-IV, scheduled for elective surgery
- **Exclusion criteria**
  - Patients with an abnormal Allen test
  - malformation of the forearm arteries on ultrasound
  - skin erosions
  - haematomas
  - unstable vital signs
  - emergency surgery.
- **Expected duration of study** : 1 year after IRB approval

#### Methodology


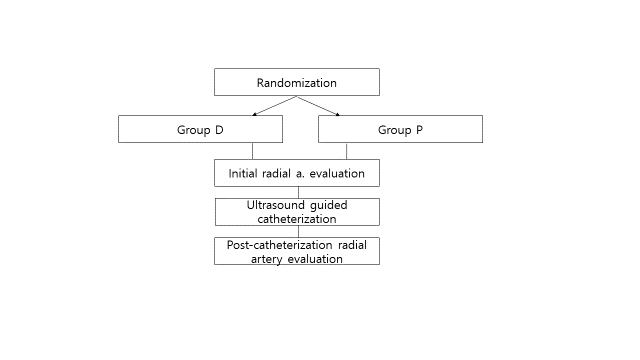


- **Study flow**

① Check the dual supply by performing Allen's test.

② After holding a towel under the wrist and fixing it in a lightly extended state, perform initial radial artery evaluation using a 5–10-MHz linear probe of an ultrasound machine.

③ Without knowing the information on ②, disinfect the radial artery cannulation area, lay a small follicle, and perform the procedure using a 20G angiocath under aseptic conditions with an ultrasound probe and a sterile sheath.

④ Time is measured from the start of catheter needle insertion using a stopwatch, and after catheter insertion, the arterial pressure line is connected and the arterial wave is confirmed on the monitor.

⑤ After completion, use ultrasound once more to check complications such as thrombosis, hematoma, edema, and vasospasm.

#### Safety Considerations

#### Moderate degree of adverse events associated with the study procedure

#### Severity of the adverse reaction is evaluated by the following criteria.

#### ① Mild: It can be tolerated easily even though it has subjective or other symptoms.

#### ② Moderate: It is uncomfortable enough to interfere with daily life.

#### ③ Severe: To be able to perform normal daily life

#### The anesthesiologist specialist JHA and JGS will perform close monitoring if the change is observed after surgery. If serious adverse events are observed, they should be reported to the IRB and treated in accordance with in-hospital treatment standards.

#### During the study period, the researcher (anesthesiologist) examines and measures and performs vital monitoring. <Confidentiality of the subject> The record of the subject's identity will be kept confidential and the identity of the subject will remain confidential even when the results of the clinical trial are published. The subject's charts and case record information are kept confidential and kept in a confidential facility and in accordance with its management standards. <Method of coding data> In all documents related to the clinical trial, such as case records, record and distinguish the patient's identification code (usually Case No.) rather than the patient's name.

#### Data Management and Statistical Analysis

- **Data** : All patient information is coded and documented.
- **Sample size calculation**

A pilot study was conducted for a total of 14 patients, 7 in each group. In elderly patients, the mean (SD) was 19 (4.7) sec when the Angiocatheter was placed using the dynamic needle tip technique, and 22 (7.4) sec in the case of using the conventional short axis view. Based on this, sample size calculation was performed bilaterally using the t-test. α err = 0.05, Power (1-β err) = 0.8 69 sample size in each group and 76 patients in each group considering 10% dropout rate. Finally, 152 patients (the same for all patients) were planned.

- **Statical analysis**
- **The primary outcome** : The success rate of arterial cannulation in the first attempt
- **The secondary outcome :** Cannulation time, the number of insertion attempts, and catheterisation-related complications

#### Quality Assurance

#### 1) Preservation of documents related to clinical trials: The institution conducting clinical trials shall preserve various data (including electronic documents) related to the conduct of clinical trials, including clinical trial plans and records on the management of clinical trial procedures, for a period of 10 years from the end of the study .

#### 2) Confidentiality: Confidentiality of data and test subject records: All test subject identification information must be kept confidential, and the case record will include the test subject number and the test subject initials. The subject will be able to view the subject's medical records for the purpose of identifying the information collected by the IRB and the Food and Drug Administration and will inform that the information will be handled in a strictly confidential manner.

#### 3) Clinical Trial Management Standards (KGCP, ICH E6): The procedures set out in this plan are based on the Clinical Trial Management Standards (KGCP: ICH E6) for testing, ) And the fundamental spirit of the Helsinki Declaration.

#### 4) Clinical trial review committee (IRB): Before starting the trial, the examiners should submit a copy of the clinical trial plan, the consent form, the data and procedures related to the recruitment of test subjects, It must be approved. The IRB's decision on the conduct of the test shall be communicated to the tester prior to commencement of the test. The person responsible for the clinical trial will report to the IRB on the progress of the trial and any serious adverse events, life-threatening problems or missions, and notify the IRB at the end of the other tests.

#### 5) Test subject's consent: The examiner should explain to the test subject (and / or his / her representative) participating in the clinical trial so that they can easily understand the nature of the test, the expected results, etc., , The test subject's written consent shall be signed and dated by the examiner and the subject and / or the agent. The original signed agreement must be kept by the tester and a copy must be given to the subject or the subject's representative. The examiner should not carry out any tests for the purpose of clinical studies before obtaining consent from the subject.

#### 6) Surveillance Survey: The purpose of surveillance surveillance surveillance surveys is to ensure that all research activities and documents related to the research are carried out in accordance with the protocol, GCP, ICH guidelines and other regulatory requirements. And to examine them systematically and independently. The relevant authorities and the Food and Drug Administration may request that the relevant documents, case records, and other test documentation be viewed for inspection or surveillance, and the tester shall permit and cooperate with this process at all times. The researcher should immediately contact the relevant department of the regulatory agency if the investigation is scheduled.

#### Expected Outcomes of the Study

The efficacy of the DNTP method can be confirmed in elderly patients over 70 years of age.

#### Dissemination of Results and Publication Policy

#### Corresponding author: Jin Hee Ahn, M.D., Ph.D

#### Duration of the Project

The study period is one year after the clinical trial approval. However, when the study is completed before that time, the time point is the end point of the study.

#### Project Management

Jin Hee Ahn : Patient collection, Manuscript writing and critical comments

Jae-Geum Shim : Study design, Data collection

#### Ethics

The investigator explained to the guardian of the patient one day before the anesthesia all protocols for this study for more than 30 minutes, and the informed consent was obtained after the patient's guardian understood it all

#### Budget

#### This research received no specific grant from any funding agency in the public, commercial, or not-for-profit sectors.

#### Other support for the Project

None.

#### Collaboration with other scientists or research institutions

None.

#### Curriculum Vitae of investigators

*Attached as additional files

#### Other research activities of the investigators

The Principal investigator (JHA) contributed over 50% to study design and overall research participation in this study.

#### Financing and Insurance

#### Financing and insurance is based on the Samsung Medical Centre compensation standard.

Researcher CV

1. 성명 : 안진희(Jin Hee Ahn)
2. 소속／직위 : 강북삼성병원 / 임상조교수
3. 전공 : 마취통증의학과
4. 생년월일 : 1982.12.11
5. 성별 : 여
6. 주소 : 서울시 송파구 풍납동 잠실올림픽공원 아이파크 103동 401호
7. 연락처 : 01093593237
8. 이메일 : [blatt.ahn@samsung.com](mailto:blatt.ahn@samsung.com)
9. 학력 : 경희대학교 의학전문대학원 석사, 박사
10 자격 : 마취통증의학과 전문의
11. 경력 : 2012.3~2013.2 경희대학교 인턴
2013.3~2017.2 삼성서울병원 전공의
2017.3~2019.2 삼성서울병원 전임의
2019.3~ 현재 강북삼성병원 임상조교수

12. 수상이력

2019.3 대한소아마취학회 우수초록상

2020.10 대한소아마취학회 우수학술상

2020.10 대한소아마취학회 최우수초록상

13. 연구 논문

**< 대표적 연구실적 요약문 >**

**1저자 및 교신저자(2019-2021)**

| 연구실적 제목 | Ultrasound-guided lung sliding sign to confirm optimal depth of tracheal tube insertion in young children. | | | |
| --- | --- | --- | --- | --- |
| 논문실적정보  □ | 게재지(저널명) | British journal of anaesthesiology | Impact Factor | 6.199 |
|  | ISSN | 0007-0912 | 게재년월 | 2019.09 |
|  | 역할(제1, 교신, 참여) | 제1저자 | 참여자수 | 5명 |

| 연구실적 제목 | Pre-administration of remifentanil in target-controlled propofol and remifentanil anesthesia prolongs anesthesia induction in neurosurgical patients: A double-blind randomized controlled trial | | | |
| --- | --- | --- | --- | --- |
| 논문실적정보  □ | 게재지(저널명) | medicine | Impact Factor | 1.870 |
|  | ISSN | 0025-7974 | 게재년월 | 2019.01 |
|  | 역할(제1, 교신, 참여) | 제1저자 | 참여자수 | 6명 |

| 연구실적 제목 | Total Intravenous Anesthesia Maintained the Degree of Pre-Existing Mitral Regurgitation Better than Isoflurane Anesthesia in Cardiac Surgery: A Randomized Controlled Trial | | | |
| --- | --- | --- | --- | --- |
| 논문실적정보  □ | 게재지(저널명) | Clinical Journal of medicine | Impact Factor | 5.38 |
|  | ISSN | 2077-0383 | 게재년월 | 2019.07 |
|  | 역할(제1, 교신, 참여) | 제1저자 | 참여자수 | 3명 |

| 연구실적 제목 | Effects of neuromuscular blockade reversal on bispectral index and frontal electromyogram during steady-state desflurane anesthesia: a randomized trial | | | |
| --- | --- | --- | --- | --- |
| 논문실적정보  □ | 게재지(저널명) | Scientific reports | Impact Factor | 4.011 |
|  | ISSN | 2045-2322 | 게재년월 | 2019.07 |
|  | 역할(제1, 교신, 참여) | 제1저자 | 참여자수 | 5명 |

| 연구실적 제목 | Improvement of laryngoscopic view by hand-assisted elevation and caudad traction of the shoulder during tracheal intubation in pediatric patients | | | |
| --- | --- | --- | --- | --- |
| 논문실적정보  □ | 게재지(저널명) | Scientific reports | Impact Factor | 4.011 |
|  | ISSN | 2045-2322 | 게재년월 | 2019.02 |
|  | 역할(제1, 교신, 참여) | 제1저자 | 참여자수 | 6명 |

| 연구실적 제목 | The frequency of gastroesophageal reflux when radiofrequency catheter ablation procedures for atrial fibrillation under general anesthesia with a supraglottic device: Observational pilot study. | | | |
| --- | --- | --- | --- | --- |
| 논문실적정보  □ | 게재지(저널명) | medicine | Impact Factor | 2.311 |
|  | ISSN | 0025-7974 | 게재년월 | 2021.02 |
|  | 역할(제1, 교신, 참여) | 제1저자 | 참여자수 | 9명 |

| 연구실적 제목 | The angle range of leg abduction with external hip rotation which can minimize femoral artery and vein overlap in pediatric patients. | | | |
| --- | --- | --- | --- | --- |
| 논문실적정보  □ | 게재지(저널명) | Pediatric anesthesia | Impact Factor | 2.037 |
|  | ISSN | 1155-5645 | 게재년월 | 2019.04 |
|  | 역할(제1, 교신, 참여) | 제1저자 | 참여자수 | 6명 |

| 연구실적 제목 | Comparison of Bispectral Index and Patient State Index values according to recovery from moderate neuromuscular block under steady-state total intravenous anesthesia | | | |
| --- | --- | --- | --- | --- |
| 논문실적정보  □ | 게재지(저널명) | Scientific reports | Impact Factor | 3.998 |
|  | ISSN | 2045-2322 | 게재년월 | 2021.3 |
|  | 역할(제1, 교신, 참여) | 제1저자 | 참여자수 | 4 명 |

| 연구실적 제목 | Effects of prophylactic atropine on the time to tracheal intubation with the pre-administration of remifentanil | | | |
| --- | --- | --- | --- | --- |
| 논문실적정보  □ | 게재지(저널명) | Aneasthesiologica Scandinavica | Impact Factor | 2.050 |
|  | ISSN | 1339-6576 | 게재년월 | 2021.3 |
|  | 역할(제1, 교신, 참여) | 교신저자 | 참여자수 | 8 명 |

| 연구실적 제목 | Text Mining Approaches to Analyze Public Sentiment Changes Regarding COVID-19 Vaccines on Social Media in Korea | | | |
| --- | --- | --- | --- | --- |
| 논문실적정보  □ | 게재지(저널명) | International journal of Environmental Research and public health | Impact Factor | 2.849 |
|  | ISSN | 1660-4601 | 게재년월 | 2021.06 |
|  | 역할(제1, 교신, 참여) | 교신저자 | 참여자수 | 6명 |

| 연구실적 제목 | Comparison of intragastric pressure between endotracheal tube and supraglottic airway devices in laparoscopic hepatectomy: A randomized, controlled, non-inferiority study | | | |
| --- | --- | --- | --- | --- |
| 논문실적정보  □ | 게재지(저널명) | medicine | Impact Factor | 1.552 |
|  | ISSN | 0025-7974 | 게재년월 | 2021.10 |
|  | 역할(제1, 교신, 참여) | 제1저자 | 참여자수 | 8명 |

| 연구실적 제목 | Utility of ultrasound evaluation of I-Gel ® placement in children: An observational study | | | |
| --- | --- | --- | --- | --- |
| 논문실적정보  □ | 게재지(저널명) | Pediatric anesthesia | Impact Factor | 2.311 |
|  | ISSN | 1155-5645 | 게재년월 | 2021.07 |
|  | 역할(제1, 교신, 참여) | 제1저자 | 참여자수 | 7명 |

| 연구실적 제목 | Machine learning model for predicting the optimal depth of tracheal tube insertion in pediatric patients: A retrospective cohort study | | | |
| --- | --- | --- | --- | --- |
| 논문실적정보  □ | 게재지(저널명) | PlosOne | Impact Factor | 2.740 |
|  | ISSN | 1932-6203 | 게재년월 | 2021.8 |
|  | 역할(제1, 교신, 참여) | 교신저자 | 참여자수 | 6명 |

| 연구실적 제목 | Gastric emptying of preoperative carbohydrate in elderly assessed using gastric ultrasonography A randomized controlled study | | | |
| --- | --- | --- | --- | --- |
| 논문실적정보  □ | 게재지(저널명) | Medicine | Impact Factor | 1.552 |
|  | ISSN | 0025-7974 | 게재년월 | 2021.9 |
|  | 역할(제1, 교신, 참여) | 제1저자 | 참여자수 | 7명 |

| 연구실적 제목 | The effect of changes in cerebral oximeter values during cardiac surgery on the incidence of postoperative cognitive dysfunction (POCD): A retrospective study based on propensity score matched analysis | | | |
| --- | --- | --- | --- | --- |
| 논문실적정보  □ | 게재지(저널명) | PlosOne | Impact Factor | 2.740 |
|  | ISSN | 1932-6203 | 게재년월 | 2021.12 |
|  | 역할(제1, 교신, 참여) | 제1저자/교신저자 | 참여자수 | 8명 |

**공저자(2019-2022)**

| 연구실적 제목 | Comparison of vasodilatory properties between desflurane and sevoflurane using perfusion index: a randomised controlled trial | | | |
| --- | --- | --- | --- | --- |
| 논문실적정보  □ | 게재지(저널명) | British journal of anaesthesiology | Impact Factor | 6.880 |
|  | ISSN | 0007-0912 | 게재년월 | 2020.12 |
|  | 역할(제1, 교신, 참여) | 공저자 | 참여자수 | 5명 |

| 연구실적 제목 | Evaluation of ultrasound-guided erector spinae plane block for postoperative management of video-assisted thoracoscopic surgery: a prospective, randomized, controlled clinical trial | | | |
| --- | --- | --- | --- | --- |
| 논문실적정보  □ | 게재지(저널명) | Journal of thoracic disease | Impact Factor | 2.046 |
|  | ISSN | 2077-6624 | 게재년월 | 2020.8 |
|  | 역할(제1, 교신, 참여) | 공저자 | 참여자수 | 5명 |

| 연구실적 제목 | Application of machine learning approaches for osteoporosis risk prediction in postmenopausal women | | | |
| --- | --- | --- | --- | --- |
| 논문실적정보  □ | 게재지(저널명) | Archives of Osteoporosis | Impact Factor | 2.017 |
|  | ISSN | 1862-3522 | 게재년월 | 2020.12 |
|  | 역할(제1, 교신, 참여) | 공저자 | 참여자수 | 5명 |

| 연구실적 제목 | Efficacy of extracorporeal shockwave therapy in the treatment of postherpetic neuralgia: A pilot study | | | |
| --- | --- | --- | --- | --- |
| 논문실적정보  □ | 게재지(저널명) | Medicine | Impact Factor | 1.552 |
|  | ISSN | 0025-7974 | 게재년월 | 2020.3 |
|  | 역할(제1, 교신, 참여) | 공저자 | 참여자수 | 5명 |

| 연구실적 제목 | Comparison of single minimum dose administration of dexmedetomidine and midazolam for prevention of emergence delirium in children: a randomized controlled trial | | | |
| --- | --- | --- | --- | --- |
| 논문실적정보  □ | 게재지(저널명) | Journal of anesthesia | Impact Factor | 1.628 |
|  | ISSN | 1438-8359 | 게재년월 | 2020.02 |
|  | 역할(제1, 교신, 참여) | 공저자 | 참여자수 | 5명 |

| 연구실적 제목 | Preoperative Oral Carbohydrate Loading in Laparoscopic Gynecologic Surgery: A Randomized Controlled Trial | | | |
| --- | --- | --- | --- | --- |
| 논문실적정보  □ | 게재지(저널명) | Journal of Minimally Invasive Gynecology | Impact Factor | 3.107 |
|  | ISSN | 1553-4650 | 게재년월 | 2021.05 |
|  | 역할(제1, 교신, 참여) | 공저자 | 참여자수 | 6명 |

14. 임상시험·임상연구 수행 이력

15. 임상시험등 종사자교육·생명(연구)윤리교육 이수 이력

2020.12.11 임상시험 종사자교육

16. 작성일자 : 2022.03.03
